# A Parsimonious Approach to Modeling Animal Movement Data

**DOI:** 10.1371/journal.pone.0004711

**Published:** 2009-03-05

**Authors:** Yann Tremblay, Patrick W. Robinson, Daniel P. Costa

**Affiliations:** 1 Institut de Recherche pour le Development, CRH UMR 212, Sète, France; 2 Long Marine Laboratory, University of California Santa Cruz, Santa Cruz, California, United States of America; University of Exeter, Cornwall Campus, United Kingdom

## Abstract

Animal tracking is a growing field in ecology and previous work has shown that simple speed filtering of tracking data is not sufficient and that improvement of tracking location estimates are possible. To date, this has required methods that are complicated and often time-consuming (state-space models), resulting in limited application of this technique and the potential for analysis errors due to poor understanding of the fundamental framework behind the approach. We describe and test an alternative and intuitive approach consisting of bootstrapping random walks biased by forward particles. The model uses recorded data accuracy estimates, and can assimilate other sources of data such as sea-surface temperature, bathymetry and/or physical boundaries. We tested our model using ARGOS and geolocation tracks of elephant seals that also carried GPS tags in addition to PTTs, enabling true validation. Among pinnipeds, elephant seals are extreme divers that spend little time at the surface, which considerably impact the quality of both ARGOS and light-based geolocation tracks. Despite such low overall quality tracks, our model provided location estimates within 4.0, 5.5 and 12.0 km of true location 50% of the time, and within 9, 10.5 and 20.0 km 90% of the time, for above, equal or below average elephant seal ARGOS track qualities, respectively. With geolocation data, 50% of errors were less than 104.8 km (<0.94°), and 90% were less than 199.8 km (<1.80°). Larger errors were due to lack of sea-surface temperature gradients. In addition we show that our model is flexible enough to solve the obstacle avoidance problem by assimilating high resolution coastline data. This reduced the number of invalid on-land location by almost an order of magnitude. The method is intuitive, flexible and efficient, promising extensive utilization in future research.

## Introduction

Monitoring the movement of animals is fundamental for investigating processes and patterns of animal distribution, habitat use and selection, habitat connectivity, recruitment, migrations, and foraging strategies. Movements of freely ranging animals are typically studied using some form of telemetry due to the difficulties of visually tracking individual animals in the wild. However, the various forms of telemetry come with certain limitations, such as limited spatial accuracy and low and/or uneven temporal resolution of recorded locations [1]–[6]. For example, satellite tracking using the ARGOS system yields location estimates that are categorized into six different quality classes (i.e. different errors), with uneven sampling due to the availability of the satellites overhead coupled with the animals' behavior and location [6], [7].

A variety of approaches have been developed to correct telemetry data by: 1) reducing spatial errors and 2) correcting for temporal lags and unevenness between data points. For the first process, filtering techniques are commonly applied to the data, based on the previous estimation of a maximum traveling speed of the animal [6], [8], [9], and eventually an additional angle filter [10]. Such filters remove unlikely locations, but they also increase the temporal gap between analyzed locations. For the second process, interpolation between known locations is performed to provide equally spaced locations for a given time interval [2]. These processes are appropriate for large scale studies, but they prevent us from using some potential positional information from discarded locations and the known spatial inaccuracy of the data is ignored [5]. Usually, 30% (+/−20%) of ARGOS locations are discarded by filters (Coyne, pers. com.) but this can reach up to ∼50–75% in some instances [1] (this work). However, these processes have the advantages of being intuitively easy to understand and generally quite simple to implement.

State-space modeling (or state-space models; SSM) is an alternative process that uses the error in the data as a source of information to infer the likelihood of the animal's position [5], [11]. In these models, a mechanistic model of movement is coupled to the data, and a probability of presence at a certain point is inferred based on the estimated state of the animal. The process model predicts the future state of an animal given its current state [12]. Observation errors are included in the probability calculations, as well as other information if they are available, such as sea-surface temperature [13]. These models are complicated to both understand and implement, often forcing ecologists researching animal movement to team up with statistical modelers for their development and analysis [12]. More importantly, SSM is not a uniform framework: state-space models can use “extended” or “unscented” Kalman filters [13], “hidden Markov” processes [14], “particle filters” [15], and discussion continues over which method to use [16]. Even more importantly, SSM are claimed to be robust [5] and better than classical techniques [12], but to our knowledge, their performances have never been objectively tested with tracking data from wild animals because by definition we only know where an animal is through the imperfect method used. However, a recent study has attempted to gain insight into SSM performances [17], and has revealed that like other methods SSM have some limitations (discussed later in this work). In addition, though rarely considered, SSM often require extremely long processing times (S. Jorgensen, H. Bailey, pers. *comm.*).

The aim of this paper is to propose a simpler, non-state-based random walk (RW) modeling approach that uses forward particle sampling as a parsimonious, intuitive, efficient and practical alternative to correcting and interpolating tracking data.

In developing this new methodology, we imposed several requirements:

1. As in state-space models, we use estimates of spatial accuracy as a source of information to infer a probable animal position for a given time.
2. Contrary to state-space models, we do not speculate on the unknown state of an animal to infer a subsequent position.
3. The method must output a track with a custom fixed time interval, thus dealing with the corrective and interpolating processes in a single step.
4. In many cases, other information independent from the tracking data can also be used in the modeling process, such as physical boundaries known to constrain dispersal or habitat characteristics known to provide more or less favorable habitat. For example, these can be forest or city limits in terrestrial environment, or coastlines in the marine environment. We must have a way to include these sources of information in the process.
5. Each output estimate of the animal's position must come with a valid estimation of confidence.
6. The method must be tested on real data in a way that performance can be validated.
7. Each step must be intuitively easy to understand, i.e. as simple as possible. While this is subjective, it will be crucial in determining both the usefulness and probability that the method will become accepted and employed by the greater animal tracking community.

## Materials, Methods and Results

### Ethics Statement

All procedures used were approved by the UCSC CARC (IACUC) committee and permitted under NMFS marine mammal permits #786-1463 and #87-143.

### Data Collection

Our focus here is on tracking data collected via the ARGOS satellite system and archival light-based geolocation telemetry, which are the two major techniques requiring post-processing of raw data. The general framework is, however, not restricted to these tracking techniques and can be adapted to any tracking data. Few studies have evaluated the performance of a model for tracking data because the true position of animals (at sea) has until recently been impossible to determine with better accuracy than with the actual tracking method used. To validate our method, we considered several movement pathways from marine animals, each bringing a possibility of evaluating the accuracy of the method and/or posing a particular analytical challenge, as detailed below:

1. Dataset 1
  The first data set was composed of 3 Argos tracks of adult female northern elephant seals, *Mirounga angustirostris* (Gill, 1866) during their post-breeding migrations. The tags used were ARGOS-linked GPS tags (SMRU GPS-SRDL, i.e. ARGOS+GPS). For battery management purposes, the tags were duty-cycled for the recording of GPS positions (1 day on, 3 days off) whereas ARGOS transmissions were continuously obtained. By nature, GPS data are much more accurate than ARGOS data (estimated unpublished errors: ∼5–60 m *vs.* ∼150–10000 m respectively), so we used these positions as our reference locations. The three tracks had distinct qualities (Table 1), so we were able to evaluate the effect of initial data quality on the performance of our model. For this dataset, the model was run using ARGOS data and the results were compared to the GPS data.
2. Dataset 2
  The second dataset is one ARGOS track from one adult female northern elephant seal. This animal was equipped with an ARGOS-only transmitter (SMRU SRDL). Although no GPS data were available for comparison, this track was chosen because the animal ventured into coastal waters of British Colombia (Canada), into a meander of fjords and islands. Elephant seals do not cross islands and do not haul-out during their migrations. Therefore, this track must be constrained by coastlines. We used this knowledge by assimilating the Global Self-consistent Hierarchical, High-resolution Shoreline database [18] in our model. This coastline dataset comes in 5 different resolutions from “crude” to “full”. We used the “high” resolution dataset (Max. error = 200 m). For this dataset, the model was run using ARGOS data and assimilated coastline data and the results were visually compared to the distribution of land masses.
3. Dataset 3
  The third dataset is a track obtained from one of the three female elephant seals from Dataset I, but in this case her track was estimated using the geolocation method. Diurnal patterns of light levels measured via a time series are used to estimate one position per day [19]. This method is less accurate than either the ARGOS or the GPS technique (estimated error ∼50–700 km) and can be improved in marine environments by assimilating remotely-sensed sea-surface temperature grids [3], [13], [20]. A Time-Depth Recorder carried by the animal (TDR, MK9 Wildlife Computers, Redmond, USA) recorded hydrostatic pressure, water temperature and ambient light levels. The geolocation algorithm from Wildlife Computers (Software WC_GPE version 1.02.0005) was used to calculate geographic positions from the light level time series. The IKNOS-DIVE program (Tremblay, unpublished) was used to analyze diving behavior as well as to extract oceanographic parameters such as sea-surface temperature (SST) for each recorded dive. Only SST values were considered in this work. We assimilated the time series of known SST from the animal with matching daily remote-sensing SST 11 km-grids consisting of a blended product of merged SST information in order to reduce cloud cover error [21]. This remote-sensing dataset was produced by the NOAA CoastWatch Program, the NOAA NESDIS Office of Satellite Data Processing and Distribution, the NASA's Goddard Space Flight Center, and OceanColor Web (details: http://coastwatch.pfel.noaa.gov/infog/BA_ssta_las.html). The 11 km grain of these data is fine with respect to the geolocation accuracy (at least by a factor 10), and therefore they are appropriate for this analysis. This track was validated using the same GPS track described in Dataset 1. For this dataset, the model was run using geolocation data and assimilated sea-surface temperatures, and the resulting locations were compared to the GPS positions.

**Table 1:** Parameters defining the qualities of the 3 elephant seal ARGOS tracks from dataset 1 (see methods).

Track number	#1	#2	#3
Duration (Days)	83.8	68.3	222.2
Number of raw locations	988	652	1042
Number of raw locations per day	11.8	9.5	4.7
ARGOS Class 3 (%)	0.2	0	0.4
ARGOS Class 2 (%)	1.2	0.6	0.7
ARGOS Class 1 (%)	2.9	1.5	2.2
ARGOS Class 0 (%)	11.0	7.4	8.7
ARGOS Class A (%)	29.9	23	22.7
ARGOS Class B (%)	40.4	49.5	53.4
ARGOS Class Z (%)	14.4	17.9	11.8
Percentage of locations removed by speed filter	52.8	64.4	55.0
Number of filtered location per day	5.6	3.4	2.1

Tracks are sorted by decreasing order of quality (based of the number of location per day). The number of location for the different ARGOS classes is given as percentages.

Field methodologies followed standard and approved procedures by the Institutional Animal Care and Use Committee at the University of California (Santa Cruz) and were described elsewhere (see [22]). All available ARGOS locations were used including class Z and secondary locations.

### Basic principles of the model

Animal movement is best described as a time series of movement steps [23]. Each step is characterized by an azimuth (or bearing) and a distance between two distinct points in time, which in turn determines a speed value. The distributions of the azimuth and speed values in a series of steps will determine the type of movement of the animal, from a straight line to a Brownian (random) motion. This principle can be used to generate artificial tracks of known characteristics, by randomly selecting consecutive azimuth and speed values from distributions of controlled characteristics [17], [24], [25]. Similarly, our model recreates many possible tracks using distributions of speed and azimuth controlled by the raw data and their associated error distributions.

**Figure 1:**
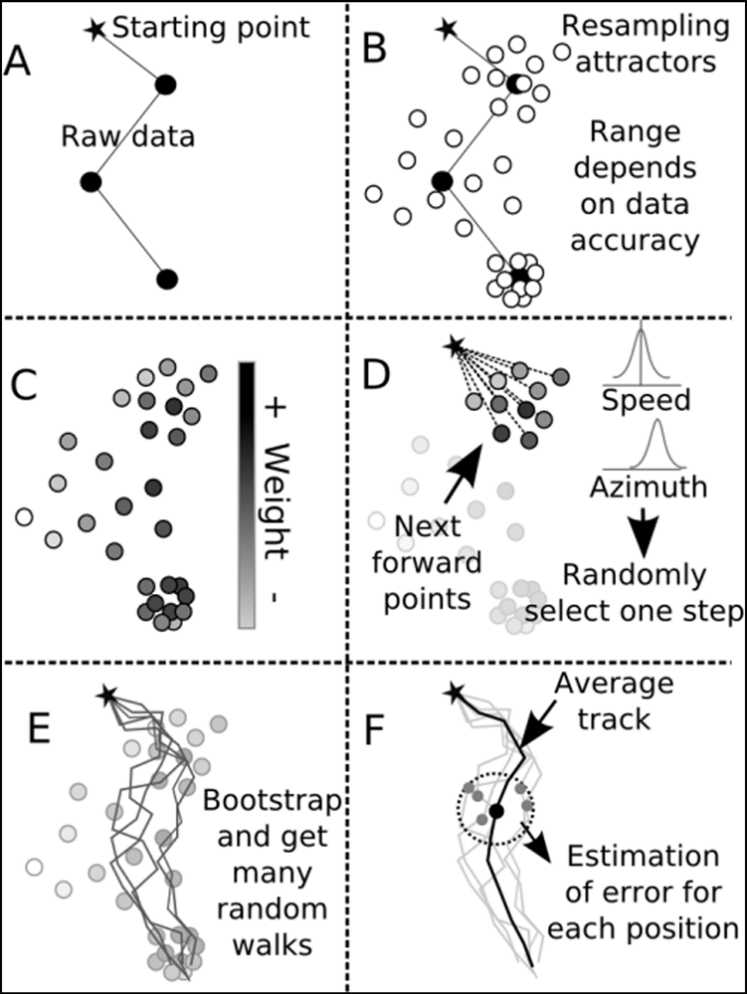
Description of the steps for the forward particle filtering model. Based on the raw data (A) and knowledge about their inaccuracy, the first step consists of generating a number of possible locations for each recorded point (B). The distribution of these particles follows a known or estimated error distribution for each point. Based on the likelihood of the speed required to get from a point to a given particle or any other known information if relevant, a weight is assigned to each particle (C). From a starting position, some forward particles will serve as attractors for constructing random walks. These forward particles define a distribution of speed and azimuth from which one random step is selected (D). The repetition of this process generates one random walk. This process is bootstrapped in order to generate many possible random walks (E). From this set of random walks, an average track is calculated. For each position of the average track, an error can be estimated from all of the corresponding locations of the set of random walks (F).

**Figure 2:**
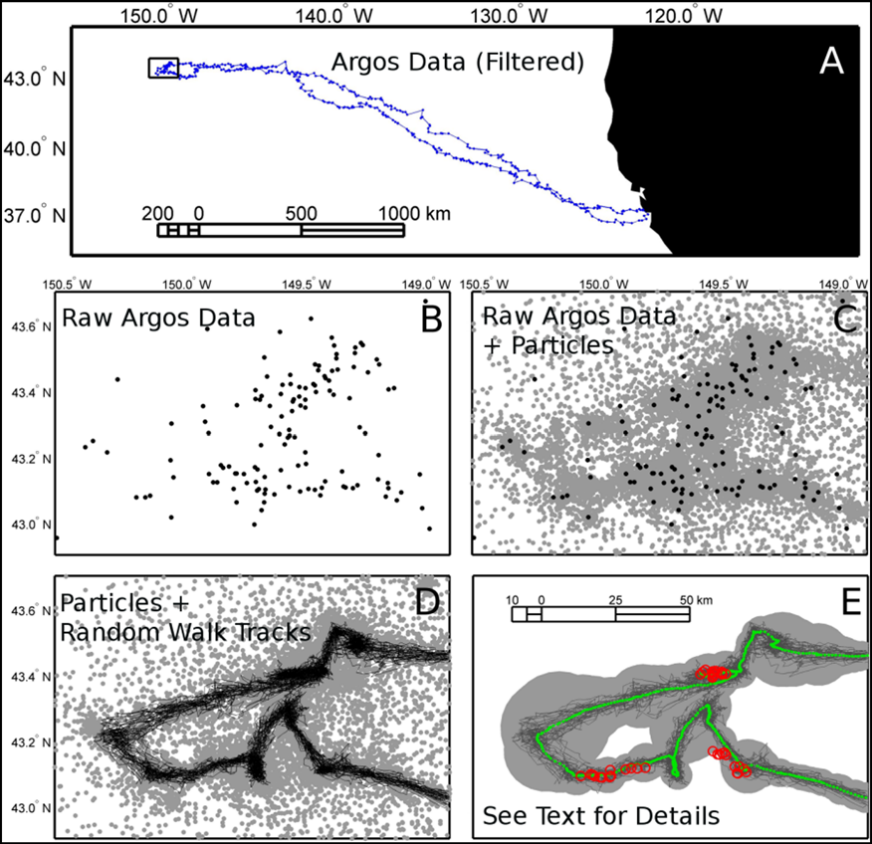
Illustration of the various steps of the forward particle sampling random walk model on one ARGOS track of a northen elephant seal from Año Nuevo, California, USA. Panel A shows the whole track as obtained using a classical speed filter and the location of the inset panels (black rectangle). From the recorded raw data (B) a set of particles is generated (gray dots in C). Using these particles a number of random walks is computed (D), which allow the calculation of an average track (green line in E) and associated error footprint (gray area in E: the accumulation of all error circles for every steps in the model). Red circles in panel E are the location of highly accurate GPS location obtained on a duty cycle fashion for this track.

The model is illustrated in figures 1 and 2. Each recorded location (Fig. 1a, 2b) can be considered the geographic average of many possible positions spread around it, depending on spatial error characteristics (Fig. 1b, 2c). Each of these possible locations (or “particles”) can then be weighted based on proper characteristics (i.e. matching recorded SST value or being on land) and/or external characteristics (i.e. feasibility of speed required to reach a particle from a given location) (Fig. 1c). We used these particles to generate the weighted distributions of azimuth and speed from which random steps were selected (Fig 1d). At each step, a new distribution of azimuth and speed is computed using the next *x* particles in the record, and one value of azimuth and speed is randomly selected and used to create the next position. If the spatial error is large, the azimuth and speed distributions widen and *vice-versa* (Fig. 1e, 2d). The output of our method is *n* time series (i.e. complete tracks), each corresponding to one bootstrapped track iteration. The “best track” can then be computed as the geographic average of the bootstrapped tracks. This leads to the possibility of estimating an error (or confidence) for each step of the average track using the dispersal of time-matching alternative positions (Fig. 1f, 2e).

### Model implementation, performance and effect of data quality (ARGOS, dataset 1)

To create the particles, we used estimated errors from our own static tests of ARGOS data [25] rather than errors given by the ARGOS system. Although the errors derived from static tests might not be the same as what would be obtained from deployed tags, we believed that they were the best estimate available. Each recorded location was resampled into 50 particles randomly selected using a bivariate normal distribution of distance (with μ = 0 and SD = error). The number of particles (here 50) was empirically fixed so that they produce a near-uniform circular distribution of azimuths around each recorded location.

With this dataset, particles were weighted according to a probability distribution of the local speeds estimated from the four prior and four following recorded points. From these locations, all combinations of speed were calculated, and only the likely ones (below a maximum speed threshold set by the user are kept, here 12.6 km/h in accordance with previous study [22]. A normal distribution with mean and standard deviation corresponding to these speeds was then used for weighting. Therefore, particles involving speeds often used by the animal (locally to each point) will have a relatively higher chance of being selected.

One characteristic of tracking data (including ARGOS) is that the error of recorded location is probabilistic. That is any location has a low probability of being very wrong, independently of its given error (i.e. errors are strongly non-gaussian [5]). This is why some presumably good ARGOS locations (with quality class of 1 for example) are sometimes found kilometers away from any reasonable position [1]. This implies that the model cannot blindly trust the accuracy of each point. In order to account for this, the azimuth and speed distributions used at each step were derived from the next 50×3 particles instead of being derived from the next 50 particles (arbitrarily). This process has a slight smoothing effect and helps the model overcome the problem of individual mistrust of recorded locations.

In our simulations, we generated random walks with steps every 30 minutes, which is close to the average duration of a dive in elephant seals [22].

**Figure 3:**
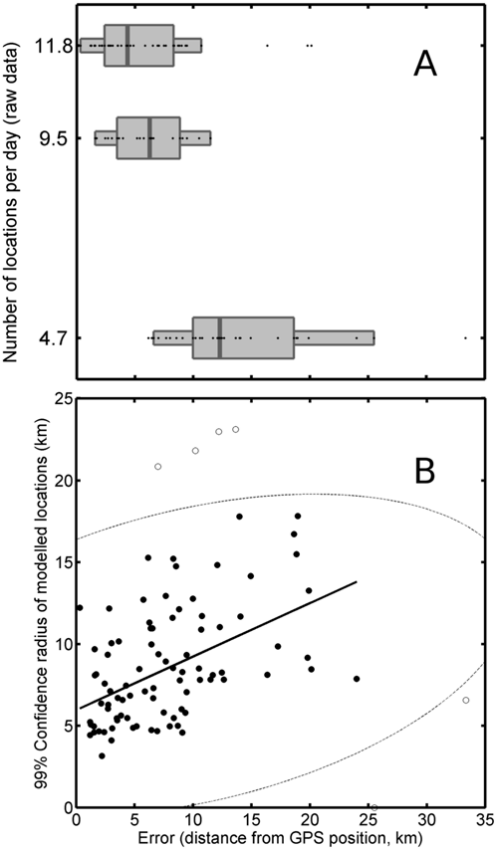
Distribution of errors (i.e. distance between the locations obtained with the model and the corresponding GPS locations) in relation to the initial number of recorded locations in the track (panel A). Box plot depicts the 25^th^ and 75^th^ percentile around the median with whiskers extending to the last non-outlier value. Outliers are observations (dots) that lay over 1.5 times the inter-quartile range from the start or end of the 25^th^–75^th^ percentile box. Panel B shows the relationship between the model estimate of confidence (99% confidence radius of the modeled positions) and the actual error. The relationship was only calculated using points within the 95% confidence ellipse.

Track quality is not easily defined because it is a combination of location quality and frequency in relation to the animal's speed, and it can be variable within a track record. In our case, reported location qualities were roughly similar in the three tracks (Table 1). Location frequency in recently obtained data in elephant seals are around three post-filtered locations per day on average [2], [26]. Therefore, the track qualities of track 1, 2 and 3 were above, equal and below average respectively (Table 1). This gives us the possibility of testing the effect of track quality on the accuracy of the model. In order to do so, we extracted all model locations that were within 2 minutes of a recorded GPS location. This yielded 41, 24 and 31 pairs of locations for the 3 tracks respectively. Fifty percent of the errors (i.e. distances between GPS locations and corresponding model-estimated locations) were below 4.0, 5.5 and 12.0 km and ninety percent of errors were less than 9.0, 10.5 and 20.0 km in track number 1, 2 and 3 respectively. Logically, errors increased with decreasing track quality (Fig. 3a).

In order to evaluate the difference between our model and classical methods, we applied a speed filter at 12.6 km/h on the raw data [22]. Filtered tracks were then interpolated every 30 minutes using a Bézier curve with mu = 0.1 [2]. A Bézier curve interpolant does not introduce additional error compared to linear interpolation, and it was shown to be a more realistic representation of movement in a fluid environment [2]. This interpolated track was compared to the GPS locations in the same way. Fifty percent of the errors were below 5.0, 13.0 and 19.5 km, and ninety percent of errors were less than 20.0, 20.5 and 41.0 km in track numbers 1, 2 and 3 respectively. Results confirm that the speed filter+interpolation method is also sensitive to the track quality [2] and show that our model reduced the positional errors by about 39% for the 50 percentile and about 52% for the 90 percentile.

**Figure 4:**
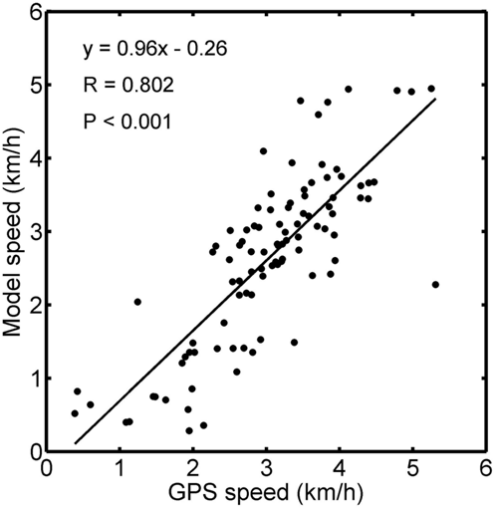
Relationship between the smoothed speed obtained with the GPS tags and the smoothed speed obtained with the model for the 97 locations that were within 2 minutes apart. Smoothing was done using a moving average including the previous and next 3 points.

Consistent with relatively low error locations, instantaneous speeds calculated from our model were positively related to the speed recorded with GPS for all locations that were less than 2 minutes apart (R = 0.638, P<0.001, N = 97). Such correlation at a small time scale is remarkable given the relative scarcity of ARGOS data. Smoothing the pattern of speed by taking into account the previous and subsequent 3 points in the record (using a moving average) permits to look at speed at a slightly coarser scale and shows further improvement of the fit, yielding a quasi one to one relationship (Fig. 4).

For each average location estimated with the model (i.e. each averaged step) we calculated the 99% confidence radius using the 30 location alternatives. This radius defines a circle that can be used as a standard measure of spatial dispersion. We showed that 75.6, 66.7, and 39.7% of the GPS positions fell within the circle in the 3 tracks respectively. By doubling the radius size, we found that 92.7, 91.7 and 87.1% of the GPS locations fell in the circle footprint. We therefore suggest that twice the 99% confidence radius calculated on the model output can be used as a valid estimate for the “real” 90% confidence error for each step. However, we found that the footprint made by the successive 99% confidence radii was usually larger than the footprint made by the random walks (Fig. 2e). This apparent contradiction was due to the fact that errors were larger along the animal path and smaller laterally (data not shown) suggesting that fitting an ellipse to the points could be a better (but more complicated) way to describe errors.

The 99% confidence radius was positively related to the actual distance between the model average location and the corresponding GPS location (Radius = 6.0+0.33×Distance, n = 87, R = 0.491, P<0.001, Fig. 3b).

**Figure 5:**
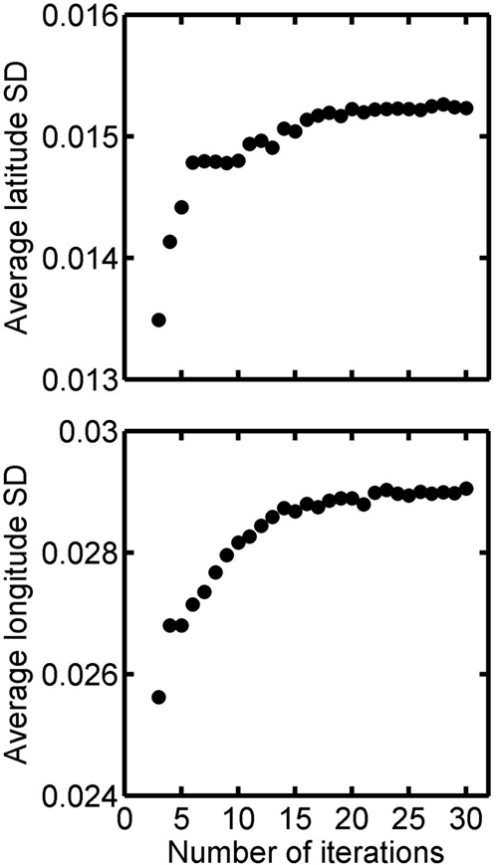
Averages of standard deviations of latitude and longitude estimates obtained with an increasing number of random walks (Number of iterations of the model).

We ran a sensitivity analysis to assess how many random walks were necessary to obtain a reliable and stable track estimate. Convergence was reached when the average standard deviations of latitude and longitude became stable, indicating that any additional track would not change the location or spatial extent of the track. This was achieved using between 15 and 20 track iterations (Fig. 5), thus the optimal ratio of computing time to track quality is achieved with about 20 iterations. The computing time required to run 20 random walks with one step every half an hour for one day (960 step calculations) required about 45 seconds of computing time, using Matlab and a laptop computer with a 2.16 Ghz dual core processor. This is about 20 times longer than “classical” speed filtering, but this is still a reasonable amount of time.

### Coastline integration (Argos, dataset 2)

**Figure 6:**
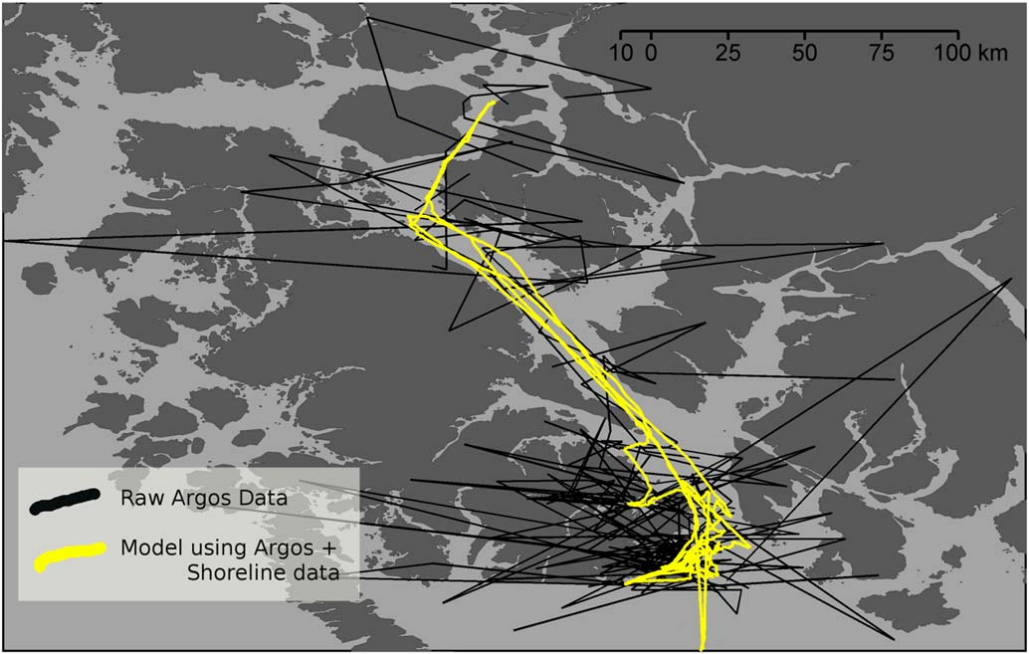
Result of the forward particle filter model applied in one northern elephant seal ARGOS track with implementation of a coast avoidance algorithm (yellow line). Black line represents the track from the raw ARGOS data. Darker polygons represent land masses and light grey background represents water. Note that only the part of the track that was within the islands (British Colombia, North East Pacific, Canada) is shown.

With Dataset 2 we confronted the problem of obstacle avoidance. In our case the seal obviously cannot cross over land, presenting a problem common in tracking studies involving coastal marine species. Solving the obstacle contouring problem in a bootstrapping context requires taking into account the compromise between a satisfactory result and the computing time needed to obtain it. Here, we added several intermediate particles to the track data in order to re-route the path when it cut through the obstacles. The geographic average of these added particles belonged to the convex Hull of the coastline polygon crossed, and were selected to achieve the shortest possible path. When several polygons were crossed, the various combinations of convex hull points were processed through Dijkstra's algorithm in order to find the shortest path between the different combinations of possible paths [27]. Each selected convex hull point was treated as a tracking point with an error arbitrarily fixed as the error of ARGOS class quality 0. Then, after re-sampling, all particles that were on land were removed (i.e. their weight = 0). With this process, some randomly generated steps can fall on land because they were not individually forced into being at sea. While this could be done, it would require considerably more computing time. However, in dataset 2, the average track from our model yields only 2.4% locations on land, whereas the speed filter+interpolation method yielded 22.6% invalid, on land locations. It is important to note that our example is an exceptionally complicated case because the spatial structure of the obstacles is both very dense and within or below the accuracy of our tracking method (Fig. 6).

### Model applied to geolocation data with Sea Surface Temperature integration (dataset 3)

Due to differences in the characteristics of light-based geolocation, we treated these data differently. In particular, errors from geolocation data are much higher latitudinally than longitudinally. The geolocation solver produced estimates of errors with some south and north boundaries for each location. We randomly generated particles so that they lay in the ellipse delimited by these boundaries. Because geolocation errors are much larger than for ARGOS data, we generated 500 particles per record instead of 50 with ARGOS data (empirically). The frequency of geolocation data provide one location per day, and SST correction applies to these locations [3], [13], [20]. However, SST at the animal is often recorded at a much finer temporal scale. For example, the time-depth recorder carried by the seal produced a measure of SST for every dive (i.e. about every 20 minutes). In order to take advantage of more of the SST data recorded, we added to the raw data a set of particles every 4.8 hours. This allowed us to use five times more information from the SST data (5×4.8 = 24 h). These particles were added in an elliptic footprint centered at an interpolated position between recorded locations and with a semi-minor axis (longitudinally) of ∼111 km (1°) and a semi-major axis (latitudinally) of ∼889 km (8°), based on published range estimates of accuracy for light-based geolocation data [13], [20].

**Figure 7:**
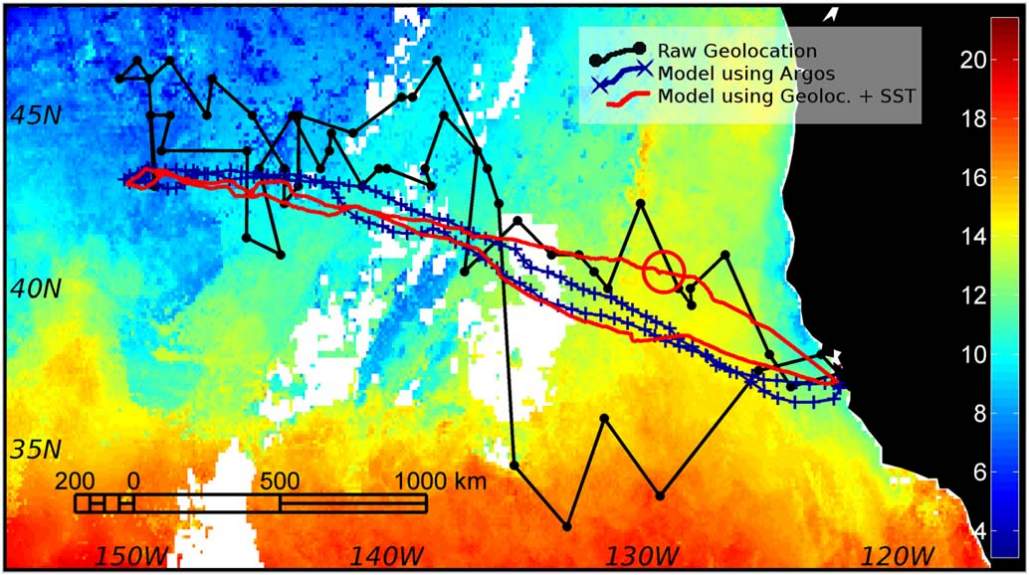
Result of the forward particle filter model applied to geolocation data in one northern elephant seal track (red line). The black line and dots represent the light-based geolocation data and the blue line and crosses represent the result of the model applied to the ARGOS data instead of the geolocation data (used here as reference since it is much more accurate – see results for details). The background colors and color bar code for the sea-surface temperature (°C) grid at the day matching the position of the large red circle. At this time and place, the SST gradient was weak, so the SST correction had little effect, thus the larger deviation of the modeled track to the reference track. Note that the GPS data were not represented because of the coarse scale of the plot and the duty cycling of the GPS data.

Fifty percent of the distances between modeled locations and the corresponding GPS locations (n = 41, locations less than 2 minutes apart) were less than 104.8 km (<0.94°) and 90% were less than 199.8 km (<1.80°). The average error was 108.4±66.8 km (0.98±0.60°). Careful examination of the track revealed that larger errors occurred at the end of the track, at locations where SST gradients were weak, and therefore SST correction had little effect (Fig. 7).

## Discussion

Bootstrapping random walks generated using forward particles in tracking data is an intuitive, relatively fast and efficient way of handling the caveats associated with current tracking techniques. To our knowledge, this is the first time that a track improvement model technique has been directly validated with animals for which “true” positions are known. Although we have illustrated our approach using ARGOS and geolocation data, the technique is applicable for any remotely sensed movement data for which an estimate of accuracy can be made.

When selecting a method, one must define an acceptable tradeoff between performance and the complexity or computation time required. In the case of tracking data, deciding on acceptable performances also depends on what is usually expected with the type of tracking methodology used For example, methods can be referred to as “ARGOS” or “Geolocation” to imply common knowledge accuracy, but both methods may give track records of very different qualities depending on the type of animal tracked and the type of tracking device used, etc. For example, diving animals may provide decreased ARGOS and geolocation track qualities compared to non-diving animals [2], [28]. Further, the type of scientific questions posed may also change the definition of “acceptable performance”. For example, long term tracking of animal migration over large scales can be accomplished with the lower accuracy geolocation method [29], [30]. Relevant to our model, the question is to know whether the gain from our method compared to classical methods is worth the effort.

### Spatial accuracy with ARGOS data

In elephant seals, very long dive durations and very short surface intervals between dives leaves few opportunities for the ARGOS tag to transmit signals to overhead satellites, which produces the poorest ARGOS track qualities [2]. Nevertheless, we showed that our model reduced spatial errors by 40–50% compared to classic speed filter+interpolation methods, with a vast majority of latent (i.e. estimated but not directly recorded) locations being within 10 km of true position. One important consequence is that the overall shape of the track was greatly improved, with removal of sudden zigzags that typically remain with speed filters. Recent GPS tracking of elephant seals confirms that these zigzags are not realistic (Costa, *unpublished*). Therefore, any analysis involving track shape such as the search for Levy flights [31], [32] or ARS patterns [24], [25] would likely be considerably enhanced by our method since spatial errors reduce the ability to discern biological signals [33]. The relationship between the error distribution and the raw track quality suggests that our model may provide even better accuracy with tracks of higher quality. This relationship is logical and proves that the amount of data available directly affects the accuracy of the model. This is because if more locations are available the model has more occasions to adjust speed along the track and therefore to use a more accurate speed distribution for generating the random walk. Although we could not quantify precisely the improvement that we obtained by assimilating coastline data, visual inspection clearly showed significant improvement as well (Fig. 6).

Our approach is based on calculating a location from a cloud of weighted particles which can be manipulated as needed. This yields great flexibility, allowing us to apply corrections based on known constraints or data available. For example, if gaps in the data exist, some particles may be added in a wide footprint within the gap, thus allowing the random walks to disperse and increasing uncertainty (i.e. decreasing confidence) where data are absent.

### Spatial accuracy with geolocation data

We showed that Sea Surface Temperature (SST) data can be assimilated into our model. To our knowledge, this is the first study reporting sub-degree average accuracy with SST-corrected geolocation data. The few studies that had assessed the accuracy of the SST-corrected geolocation method reported accuracies of 1.82°±1.54° (mean±SD) in albatrosses [3], and 1.28°±0.38° (mean±SD of means) in 4 species of fish [20]. It is important to note that these accuracies were calculated for recorded locations (i.e. in theory the best estimates) whereas our estimation of accuracy was made for latent locations. However, variance in errors is typically high because of the high variability in the quality of the light measurement and therefore comparison between studies is only vaguely informative. The take home message however is that using our model, sub-degree accuracy is achievable with latent locations if some SST gradient exists.

Interestingly, one study assimilated both SST and bathymetry in order to correct geolocation tracks of gray seals (*Halichoerus grypus*), and produced average errors of 0.85°±0.07° [28] which is slightly better than our estimates. The elephant seal that we use as an example ventured in very deep and unreachable ocean waters, and therefore, bathymetry could not possibly have improved our track. However, our method permits easy assimilation of bathymetry, by simply altering the weights attributed to each particle based on the comparison between known depth reached by the animal and the ocean bathymetry. This process is also not exclusive to SST or to any other parameter. Finally, a recently published algorithm [34] aimed at estimated positions based on light levels alone suggests that the raw data to input to our model could be substantially improved in the future, which in turn suggests that output from our model could be even further improved.

### Confidence estimates of locations

Analyzing animals' behavior in relation to environmental characteristics may enable estimation of a confidence metric for each position, which is rarely available with recorded ARGOS data (ARGOS location of class A,B and Z for example) and not obtainable with the speed filter+interpolation methods. This confidence metric is essential for interpreting the behavior of the animal at an appropriate scale. For example, if the confidence radius is 10 km, it is probably inappropriate to interpret movements shorter than this distance. Similarly, the environment characteristics at a given location may be gathered within the location error footprint rather than under each average location, potentially reducing noise in the data and improving habitat model confidence and determination.

In this paper we used a circle to estimate confidence, but this could be done differently. For example, if the errors are systematically biased towards one direction (for example latitudinally as in the case of geolocation data), it would be straightforward to determine another metric based on the output from the random walks. For example, the standard deviation in latitude and longitude, or the maximum distance between the various positions and their average could be used as well. More complex methods such as the determination of an ellipse (as mentioned earlier) could be used to account for particular spatial structure that might occur in the error footprint. For example, this could allow us to discriminate between the error vectors occurring along the animal path to the error vectors occurring laterally to the animal path. Overall, this shows that our approach is not sealed to one scheme, but instead, it is very open to user input and experimentation.

### Forward particle random walk model versus state-space model

For most applications a biased random walk approach like ours seems to be an excellent compromise between complexity, computation time, ease of implementation and effectiveness, especially when compared to state-space models (SSM). Some SSM users and developers have themselves considered the approach as a technically difficult statistical framework [12], [35]. Contrary to state-space models, we did not have to infer animal state, transition equation, measurement equation and switching model, nor did we rely upon Bayesian statistics (which is complicated for most users), and yet, we obtained very satisfactory results while preserving the possibility to use information from other sources of data, such as coastlines or SST. Other parameters, such as ocean depth, could be coupled with the animal diving depth in the same way. It is important to note that our approach cannot repeat earlier track patterns in a gap; indeed, SSM may artificially create patterns where data are sparse [17], which may be problematic for subsequent analysis.

A recent attempt to assess SSM accuracy was made using artificial tracks, and reported mean absolute errors to be at best one to two times larger than what we recorded in this study [17]. It is however unclear how our approach would compare to a state-space model approach because, to our knowledge, no state-space model application on animal movement data has been truly validated (compared to GPS locations). A direct comparison is therefore not strictly possible. What is certain is that the complexity and associated computing time is considerably reduced using our approach. A switching state-space model [36] would probably increase the computing time by a factor of 5 (Bailey, *pers. comm.*), which would amount for at least 5, 3 and 15 hours of computing to run our track numbers 1, 2 and 3 from our first dataset, respectively.

State-space models have the ability to produce an estimate of the animal's behavioral mode, and this can be seen as a major advantage over our method. The behavioral mode is usually characterized by a certain bearing (or turn) variability and a certain speed [14], [36]. It is indeed important to be able to distinguish between behavioral phases, and we believe that simple time-series analysis or other first passage time [24] or fractal landscape analysis [25] may accomplish this equally well. *A priori* there is no reason why track correction and delineation of behavioral mode must occur in the same step. In fact, it might even be simpler and more useful for researchers to compare and use several methodologies to identify behavioral states. However, this is not the topic of this work and this may require more investigations.

State-space models were presented as a way to use all available information in the tracking data without the need for filtering processes to be performed [37]. A recent study using state-space models suggested that pre-filtering of ARGOS data improves the model performance [16], and this seems to be confirmed by another recent study which used a classical speed filter prior to using the state-space model [38]. None of this is required with our approach, and at the opposite, we even used ARGOS secondary locations in addition to recorded primary locations in our model, in order to truly use the maximum information available.

Finally, it is important to note that the advantages or inconvenient of using a method or another might also depend on one's initial goals. By fitting a mechanistic model to the data, state-space models are by construction more predictive than our approach. This possibility might be of interest for comparing different mechanistic models of individual movement and therefore to explore the effects of different behavioral processes on the dispersal of individuals. This cannot be done directly with our approach, but as a second step, by comparing the output of our method to the output obtained under a given theoretical framework.

Our model uses particles, but it differs from particle sampling filters by the fact that particles are not generated using prior behavioral information [15], but instead, they are generated based on the recorded data.

### Conclusion

Previous work has shown that simple filtering is wasteful and inefficient and that additional, valuable behavioral information can be extracted from tracking data. To date, this has required methods that are both complicated and time-consuming, resulting in limited application and the potential for analysis errors due to poor understanding. The particle filter model outlined here attempts to improve the quality of tracking data while operating by a framework that is both accessible and efficient. This method improves the accuracy of positions and assigns an estimate of spatial error, facilitating subsequent post-hoc behavioral analyses. In order to share this method with the research community, we will establish a dedicated website to provide source codes, examples, and a manual. The package will be known as the IKNOS-WALK program.
